# FREQUENCY OF CLINVAR PATHOGENIC VARIANTS IN CHRONIC KIDNEY DISEASE PATIENTS SURVEYED FOR RETURN OF RESEARCH RESULTS AT A CLEVELAND PUBLIC HOSPITAL

**Published:** 2020

**Authors:** Dana C. Crawford, John Lin, Jessica N. Cooke Bailey, Tyler Kinzy, John R. Sedor, John F. O’Toole, William S. Bush

**Affiliations:** 1 Cleveland Institute for Computational Biology, Case Western Reserve University, Wolstein Research Building, 2103 Cornell Road, Cleveland, OH 44106, USA; 2 Department of Population and Quantitative Health Sciences, Case Western Reserve University, Wolstein Research Building, 2103 Cornell Road, Cleveland, OH 44106, USA; 3 Department of Genetics and Genome Sciences, Case Western Reserve University, Wolstein Research Building, 2103 Cornell Road, Cleveland, OH 44106, USA; 4 Department of Physiology and Biophysics, Case Western Reserve University; 5 Department of Nephrology and Hypertension, Glickman Urology and Kidney and Lerner Research Institutes, Cleveland Clinic, Cleveland, OH 44106, USA

**Keywords:** return of results, *APOL1*, ClinVar, chronic kidney disease, electronic health records, African Americans

## Abstract

Return of results is not common in research settings as standards are not yet in place for what to return, how to return, and to whom. As a pioneer of large-scale of return of research results, the Precision Medicine Initiative Cohort now known of All of Us plans to return pharmacogenomic results and variants of clinical significance to its participants starting late 2019. To better understand the local landscape of possibilities regarding return of research results, we assessed the frequency of pathogenic variants and *APOL1* renal risk variants in a small diverse cohort of chronic kidney disease patients (CKD) ascertained from a public hospital in Cleveland, Ohio genotyped on the Illumina Infinium Mega^EX^. Of the 23,720 ClinVar-designated variants directly assayed by the Mega^EX^, 8,355 (35%) had at least one alternate allele in the 130 participants genotyped. Of these, 18 ClinVar variants deemed pathogenic by multiple submitters with no conflicts in interpretation were distributed across 27 participants. The majority of these pathogenic ClinVar variants (14/18) were associated with autosomal recessive disorders. Of note were four African American carriers of *TTR* rs76992529 associated with amyloidogenic transthyretin amyloidosis, otherwise known as familial transthyretin amyloidosis (FTA). FTA, an autosomal dominant disorder with variable penetrance, is more common among African-descent populations compared with European-descent populations. Also common in this CKD population were *APOL1* renal risk alleles G1 (rs73885319) and G2 (rs71785313) with 60% of the study population carrying at least one renal risk allele. Both pathogenic ClinVar variants and *APOL1* renal risk alleles were distributed among participants who wanted actionable genetic results returned, wanted genetic results returned regardless of actionability, and wanted no results returned. Results from this local genetic study highlight challenges in which variants to report, how to interpret them, and the participant’s potential for follow-up, only some of the challenges in return of research results likely facing larger studies such as All of Us.

## 1. Introduction

### 1.1. Return of Results in Precision Medicine Research

Recent surveys have demonstrated that study participants would like their research-generated results, particularly genetics, returned [1–4]. Guidelines, albeit relatively new and still evolving, exist for return of clinically-generated genetic results [5, 6], but an equivalent does not exist for return of research-generated results. Unlike clinically-ordered genotyping or sequencing where patients are referred for genetic testing, research studies generate similar data but for a wider range of participants including healthy or pre-symptomatic patients.

Despite the lack of consensus on the value of genotyping and sequencing healthy participants as opposed to patients and family members [7–9] as well as what results should be delivered and how to deliver them [10, 11], several research studies have established return of result programs as early pioneers of this active discussion. Arguably the largest of these efforts is All of Us, formerly known as the Precision Medicine Initiative Cohort Program [12]. Akin to the very successful and popular UK Biobank [13], All of Us plans to enroll one million participants residing the United States as a research resource for a broad range of topics including genotype-phenotype studies [12]. With almost 150,000 participants, of whom 53% are racially/ethnically diverse and have donated biospecimens for genotyping and sequencing [14], All of Us is planning the return of pharmacogenomic results as well as clinically-relevant genetic variants to participants in late 2019 or early 2020 [15, 16].

### 1.2. Expected Scope of Returning Research Results

Returning clinically-generated results either to patients or physicians for even a handful of genetic variants requires substantial resources and infrastructure not yet widely available [17]. Reports of “medically actionable” variants observed in early whole-exome sequencing studies of reference sample sets or disease consortia have suggested that, depending on the kind of variant to be reported (e.g., pathogenic variant, pharmacogenomic variant), most if not all participants would receive results [18–20]. These estimates, however, are dependent on the quality and completeness of databases that house genotype relationships to human health. One of these publically available databases, ClinVar, was established in 2012 to offer a searchable centralized resource for genetic variant interpretation [21, 22].

To better understand the local landscape of return of research results, we examined the frequency of clinically-relevant variants in a small study population of chronic kidney disease (CKD) patients ascertained from a public hospital in Cleveland, Ohio [1]. CKD is a major health outcome in the United States affecting more than 13% of the general adult population [23]. The prevalence of CKD is higher among African Americans compared with other racial and ethnic groups, and the rate of progression to end-stage renal disease (ESRD) is disproportionally faster among individuals of African ancestry compared with individuals of European ancestry. To date, the only major factor associated with the observed disproportionate prevalence of CKD and rate of ESRD among African Americans compared with other groups is *APOL1* and its common genetic variants G1 (rs73885319) and G2 (rs71785313) [24–26]. In this study, consented participants had the option to take a short survey about their attitudes on participating in biobanks and their opinions on return of results [1]. Participants also had the option of donating biospecimens, which were subsequently genotyped using the Illumina Infinium Mega^EX^, a genome-wide genotyping array of approximately 2 million variants selected for genotype-phenotype studies in diverse populations [27]. The Illumina Infinium Mega^EX^ directly assays the two *APOL1* renal risk variants as well as >20,000 other variants annotated in ClinVar [21, 22], giving us the opportunity to estimate hypothetically what results might be returned and to whom in a study population of similar patients.

## 2. Methods

### 2.1. Study Population

As previously described [1, 28], patients were ascertained from the MetroHealth Medical System Division of Nephrology and Hypertension in Cleveland, Ohio under an umbrella kidney disease research protocol approved by the MetroHealth Institutional Review Board. All participating patients were provided written, informed consent. For this study, participants were asked to take a short survey [1] and donate biospecimens (blood) for DNA extraction. Participants also consented to investigators accessing their electronic health records (EHRs) for kidney disease-related research questions. The consent form stated that research results, including genetic results, would not be returned or available to the participant or the participant’s physician.

### 2.2. Genotyping and Quality Control

DNA was extracted from whole blood on the Qiagen QIAsymphony (Hilden, Germany) using standard protocols. A total of 134 DNA samples were then genotyped on the Illumina Infinium Expanded Multi-Ethnic Genotyping Array (Mega^EX^; San Diego, California) by the University Miami’s Center for Genome Technology. The Mega^EX^ is based on the Multi-Ethnic Genotyping Array (Mega), a custom Illumina Infinium BeadChip designed to facilitate fine-mapping and functional genomic discovery in diverse populations [27]. The Mega^EX^ targets ~2 million variants, including clinically-relevant variants from ClinVar, a public repository of genomic variation and its relationship to human health [21]. We downloaded the Mega^EX^ annotation files “PAGEII_WGSA_MEGA_annotations.tar.gz” updated 2018–08-24 and made available on the Population Architecture using Genomics and Epidemiology (PAGE) II [29] website (https://www.pagestudy.org/index.php/multi-ethnic-genotyping-array). The “wgsa_snp_column_description” file provided the following ClinVar-relevant information: rs number by ClinVar (clinvar_rs); clinical significance by ClinVar (clinvar_clinsig), including benign (2), likely benign (3), likely pathogenic (4), pathogenic (5), drug response (6), and histocompatibility (7); the trait or disease to which ClinVar clinical significance refers (clinvar_trait); and ClinVar review status summary, denoted as stars for no assertion criteria provided (0), criteria provided, single submitter (1), criteria provided, multiple submitters, no conflicts (2), reviewed by expert panel (3), and practice guideline (4) (clinvar_golden_stars).

Genotype calling was performed using Genome Studio, and variants were annotated using Illumina-provided annotation files. One DNA sample failed genotyping (<0.98 sample call rate), and variants with low call rates (<0.95) were removed during the initial quality control process. Genotype data for the remaining 133 samples were subject to further quality control using PLINK 1.9 [30], and three samples were removed for genetic sex discordance with EHR-recorded gender. Variants were then removed based on call rates (<0.98), deviations from Hardy Weinberg (at p<0.0001), and minor allele frequency (<0.1%). No cryptic relatedness was identified using KING [31]. Global genetic ancestry was estimated using ADMIXTURE [32] with CEU and YRI reference data to estimate ancestry for European American and African Americans (K=2). CHB reference data were added to estimate East Asian ancestry (K=3).

## 3. Results

### 3.1. Population characteristics

In general, the study population characteristics for the 130 participants with genotyping data that passed quality control reflected the demographics expected for CKD patient populations. That is, approximately half were female, and age at study enrollment ranged from 18 years to 91 years with an average age of 61.2 years. Most of the study population was African American or European American inferred from EHR clinical notes, and these were in agreement with global genetic ancestry estimates (92.98% and 97.96% concordant, respectively). As expected for an admixed population, samples with majority (>60%) West African ancestry had an average global Western European ancestry of 17% (range: 7–38%), consistent with most [33–35] but not all [36] previous reports for African Americans. Samples with majority (>60%) Western European ancestry were also admixed, but to a lesser extent with an average of 4.19% West African ancestry (range: 0.001–27%).

### 3.2. Frequency of APOL1 renal risk variants

As expected, G1 and G2 minor alleles were frequent among African American participants (0.21 and 0.12, respectively) but either absent (G1) or rare (G2; 0.01) among European American participants. African American participants were more likely to carry one (RA_1_; 36.84%) or two (RA2; 19.30%) renal risk alleles (RA) compared European Americans (RA_1_; 2.04%). G1 and G2 allele frequencies and renal risk allele distributions did not differ substantially when global genetic ancestry was considered (Table 1). The European-descent carriers of *APOL1* renal risk alleles observed here are admixed (15–20% African-descent based on global estimates).

### 3.3. Frequency of ClinVar-designated pathogenic variants

After genotyping quality control, the present dataset had 23,720 ClinVar-designated variants, of which 8,355 (35%) had at least one alternate allele in the dataset. Among these, we counted the number of ClinVar-designated pathogenic variants by ClinVar evidence level: 1) criteria submitted with multiple submitters and no conflicts in interpretation (two stars), 2) criteria submitted with multiple submitters and conflicts in interpretation or submitted with one submitter (one star), 3) no criteria submitted (no stars). A total of 19 pathogenic two-star ClinVar variants have at least one heterozygote in the present study population (Table 2). These 19 variants included Canavan disease *ASPA* rs12948217, a variant whose pathogenic A allele (c.693C>A, Y231X) is not distinguished from the benign C and T alleles by the Illumina Infinium Mega^EX^. After removing *ASPA* rs12948217, a total of 27 participants were heterozygous for at least one of the 18 pathogenic two-star ClinVar variants, and one participant was heterozygous for two pathogenic two-star ClinVar variants (Table 2). No homozygous participants were identified. As expected, most (14/18) pathogenic two-star ClinVar variants with at least one alternate allele in this study population are rare in the general population and autosomal recessive. An exception here is *HFE* rs1800562 (Cys282Tyr), a well-known variant associated with hemochromatosis. We identified total of seven heterozygotes: 1 African-descent and 6 European-descent participants (Table 2).

We note that two autosomal dominant pathogenic two-star ClinVar variants have at least one heterozygous carrier in this dataset: *MC4R* rs13447324 and *TTR* rs76992529. *MC4R* rs13447324 is a nonsense variant associated with obesity. In this dataset, the variant was identified in one European American/European-descent participant who at the time of study enrollment had an EHR-recorded body mass index of 28 kg/m^2^. Four African American/African-descent participants were heterozygous for *TTR* rs76992529, a missense variant associated with amyloidogenic transthyretin amyloidosis. While rare in a general European-descent population, *TTR* rs76992529 is less rare in African-descent populations (e.g., 1000 Genomes African reference data, minor allele frequency or MAF = 0.02) as evidenced here (MAF = 0.03; Table 2).

Compared with pathogenic ClinVar two-star variants, we identified a greater number of participants who carry at least one alternate allele for pathogenic ClinVar one-star variants (18 variants versus 33 variants). Pathogenic ClinVar variants with a no-star rating were the most prevalent in this dataset, where 205 variants have at least one identified heterozygote. Regardless of star rating, the alternate allele score ranged from 14 to 37, with an average of 25.84 (±4.05 standard deviations) pathogenic ClinVar alleles per participant. When “likely pathogenic” ClinVar variants regardless of star rating were included, the average alternate allele per participant increased to 29.95 ± 4.30 standard deviations (range: 17–43).

### 3.4. Pathogenic ClinVar variants and participant views on return of results

As we have previously reported [1], study participants were requested to complete a short, five-question survey probing opinions on participating in large-scale precision medicine research and return of research results. We asked the participants, “What type of results would you like to receive, check all that apply”, which included the possible responses “(c) information about your genes that may influence your doctor’s approach to your care (for example, they may order additional testing or consider alternative treatments or medications); (d) information about your genes that has uncertain significance and will not change the way that your doctor treats you; and (e) I do not want to receive any results” [1]. Among the genotyped participants, 35 selected only (c) and 17% were heterozygotes of one pathogenic ClinVar two-star variant (Table 3). Four (11%) of these 35 participants also had two *APOL1* renal risk variants. More participants (51) selected (d) alone or in combination with (c), and among these, 12 participants were heterozygotes for one pathogenic ClinVar two-star variant, and six have two *APOL1* renal risk alleles. For the seven participants who did not want to receive any results (e only), approximately one-third were carriers of a pathogenic ClinVar two-star variant, one-third had one *APOL1* renal risk allele (including one participant with both a pathogenic two-star variant and a renal risk allele), and one (14%) had two *APOL1* renal risk alleles.

### 3.5. Pathogenic ClinVar variants and changes in level of evidence

The ClinVar star ratings described herein rely on the PAGE II annotated files associated with the design of the Illumina Infinium Mega^EX^. Although not documented in the literature [27], it is likely the ClinVar database was queried sometime between 2014 (the year that the NHGRI-EBI GWAS Catalog was queried) and 2018 (the year the PAGE II annotation files were updated). We updated the ClinVar star annotations for the pathogenic ClinVar two-star and-one star variants in July 2019, and we note several differences. Among the 18 pathogenic two-star variants (Table 2), two (*HFE* rs1800562 and *GJB2* rs72474224) were downgraded to one-star ratings due to multiple submitters with conflicting interpretations. Among the 33 pathogenic one-star variants, 16 were upgraded to two-star pathogenic (10), two-star pathogenic/likely pathogenic (5), or two-star likely benign (1). The upgraded pathogenic variants include *LRRK2* rs34637584 associated with autosomal dominant Parkinson disease 8 and *SPINK1* rs148954387 associated with hereditary pancreatitis, both identified in one European-descent participant each. *SPINK1* rs148954387 is monomorphic in 1000 Genomes European reference populations, but present in East Asian reference populations (1000 Genomes MAF =0.003). The one heterozygote identified here is a participant with majority European global genetic ancestry (>60%) but described as Asian in the EHR clinical notes.

## 4. Conclusion

We describe here the distribution of *APOL1* renal risk variants as well as pathogenic ClinVar variants in a small diverse study population of CKD patients from a public hospital already surveyed for their attitudes on return of research results. While the patients consented to participate in this pilot study knowing that results would not be returned to them or their physicians, these data inform the complexity of returning genetic results generated in a research setting to diverse participants. Consistent with the MedSeq Project [17], which performed whole genome sequencing on cardiomyopathy patients and healthy participants, approximately 20% of the present study population would be receiving results for one to two pathogenic ClinVar two-star variants. All 130 participants would receive research results if ClinVar-designated pathogenic or likely pathogenic variants of all levels of evidence are to be returned. The average study participant here has 30 pathogenic or likely pathogenic ClinVar variants regardless of level of evidence.

Whether returning two-star or regardless of star ClinVar pathogenic variants, the preparation of the report promises to be challenging as the reports must describe complex medical genetics concepts such as inheritance patterns, age at onset, variable expressivity, and penetrance. Equally difficult to convey is the confidence of variant classification and its clinical impact, a difficulty amplified when the genotyped participants are asymptomatic or drawn from the general population regardless of health status. Also, variant classification and interpretation can change over time as more data are collected. ClinVar is not a static database [37], and information relayed in a static report issued to a participant could quickly become outdated [17]. Here, we observed that 45% of one-star pathogenic variants from an earlier version of ClinVar were upgraded to two-star pathogenic or likely pathogenic in a 2019 version of ClinVar.

As we [1] and others [38] have reported, research participants do not necessarily want all or any of the offered genetic results. In this small survey, ~34% of participants with genotypes wanted only “actionable” results while more than half (~55%) wanted genetic results regardless of impact to health or treatment. A small proportion (7.5%) did not want results returned to them, even if actionable. Pathogenic ClinVar two-star variants and *APOL1* renal risk variants were identified across all these groups, including *TTR* rs76992529 associated with amyloidogenic transthyretin amyloidosis, otherwise known as autosomal dominant familial transthyretin amyloidosis (FTA). FTA is a fatal, adult-onset disease with variable penetrance. Treatments are available that prevent damage from or slow the progression of amyloid deposition associated with disease; no treatments are known to reverse damage underscoring the importance of a timely diagnosis for this rare disease.

Whether or not the participant chooses to receive genetic results from research, response or action based on returned results will require resources that are not equally accessible or distributed across the US healthcare system. Medical action will require interfacing with a primary healthcare professional who may not be familiar or comfortable with the returned genetic results, including pharmacogenomic results [39, 40]. Referrals to nearby medical geneticists, genetic counselors, and other specialists may not be possible, depending on where the participant resides or depending on the participant’s ability to afford these clinical encounters and likely re-testing in a CLIA-certified laboratory. The present study participants were ascertained from a public hospital, and the majority of its patient population reside within Cleveland zipcodes associated with high poverty rates. A high proportion of patients at this hospital are on Medicaid (50% as of 2015), and all CKD patients on dialysis regardless of income level are on Medicare.

The present study has several limitations and strengths. The main limitation is sample size and the use of a genotyping array, both of which impact the ability to detect a wider range of rare, potentially actionable variants that larger genotyped or sequenced cohorts like All of Us will encounter. Another major limitation is the lack of in-depth clinical data available to assess the genotype’s relationship to the phenotype. Demographic and clinical data were extracted from participants’ EHRs for studies related to kidney disease, and these limited data are insufficient to assess the penetrance of any of the autosomal dominant pathogenic variants identified here. Even with access to the full clinical record of these patients, previous studies using EHRs to assess penetrance or to establish clinical genotype-phenotype relationships [41, 42] have highlighted challenges owing to missingness, bias, and other limitations inherent to these clinical records [43].

A major strength of the present study is its diversity. The present study population is nearly proportionally equal between African-descent and European-descent participants whereas the extent of precision medicine research and data available for these two major US groups is not [44]. Research efforts focused on non-European populations are needed and underway to ensure that informative return of results reports are available to as many study participants as possible [14, 45, 46]. Another unique feature of the present study is it is neither drawn from a general population nor from a clinically-indicated population referred for genetic testing. Participants in this study had CKD and were genotyped on the Illumina Infinium Mega^EX^ mainly for their *APOL1* status and other genome-wide variants possibly associated with kidney disease and related conditions. Approximately 60% of this CKD patient population carried one or two *APOL1* renal risk alleles, which although considered only “risk variants” by ClinVar are the subject of much discussion in return of results given that the presence of two renal risk alleles increases risk for CKD as well as possibly impacts kidney transplantation success [47]. One fifth of this study population overall were carriers of a pathogenic two-star ClinVar, five of whom also have an *APOL1* renal risk allele. For the majority of these patients, *APOL1* status or other rare variants associated with their kidney disease [48] are arguably more relevant to their immediate health, and like other healthy and patient populations receiving results, it is unclear what potential benefits would be realized if additional genetic data were returned to this already burdened patient population.

## Figures and Tables

**Table 1.** Renal risk variant distribution, by population. Counts (proportion) of study participants with no, one, or two renal risk alleles (RA), by race/ethnicity inferred from EHR clinical notes (African American or European American) and global genetic ancestry (>60% African or European-descent). Renal risk alleles are alternate alleles for either *APOL1* G1 (rs73885319), G2 (rs71785313), or both.

	RA_0_	RA_1_	RA_2_
**African American (n=57)**	25 (43.86%)	21 (36.84%)	11 (19.30%)
**African-descent (n=62)**	25 (40.32%)	24 (38.71%)	13 (20.97%)
**European American (n=49)**	48 (97.96%)	1 (2.04%)	0 (−)
**European-descent (n=63)**	61 (96.83%)	1 (1.59%)	1 (1.59%)

**Table 2.** Number of carriers of ClinVar variants with multiple submitters, no conflicts, by population. Shown are the counts of observed alternate alleles for pathogenic ClinVar two-star variants assayed by the Illumina Mega^EX^ and included in the PAGE II study annotation files. Counts are stratified by race/ethnicity inferred from EHR clinical notes (African American or European American) and global genetic ancestry (>60% African or European-descent). Also shown are variant genomic location (Genome Reference Consortium Human Build 37), alleles, rs number. disease or trait associated with the variant, inheritance pattern, and ClinVar golden star rating as of 2019. Multiple traits can be reported for a single rs number and are delineated by |. Abbreviations: African American (AA), African-descent (A), autosomal dominant (AD), autosomal recessive (AR), chromosome (chr), European American (EA), European-descent (E).

Chr:Position	Alleles	rs number	ClinVar Trait (Inheritance)	ClinVar Golden Star Rating 2019	AA Carriers (A)	EA Carriers (E)
1:21890632	G>A	rs121918007	Infantile hypophosphatasia (unknown)	2	0/56 (0/62)	0/50 (1/63)
1:45797228	C>T	rs36053993	MYH-associated polyposis| not provided| Hereditary cancer-predisposing syndrome| (AR)	2	1/56 (1/62)	0/49 (0/62)
1:76226846	A>G	rs77931234	Medium-chain acyl-coenzyme A dehydrogenase deficiency| not provided (AR)	2	0/56 (0/62)	2/50 (2/63)
1:1212453	G>A	rs121434346	Neutral 1 amino acid transport defect (AR)	2	0/56 (0/62)	0/50 (1/63)
6:26093141	G>A	rs1800562	Hemochromatosis type 1 (AR)	1	2/56 (1/62)	3/50 (6/63)
9:34647855	C>T	rs111033690	Deficiency of UDPglucose-hexose-1-phosphate uridylyltransferase| not provided (AR)	2	1/56 (1/62)	0/50 (0/63)
9:111662096	A>G	rs111033171	Familial dysautonomia (AR)	2	0/56 (0/62)	1/50 (1/63)
11:64527223	G>A	rs116987552	Glycogen storage disease, type V| not provided (AR)	2	0/56 (0/62)	1/50 (1/63)
11:66293652	T>G	rs113624356	Not provided| Bardet-Biedl syndrome (AR)	2	0/56 (0/62)	1/50 (1/63)
11:68701332	C>A	rs145226920	Spinal muscular atrophy, distal, autosomal recessive, 1 (AR)	2	0/56 (0/62)	1/50 (1/63)
13:20763612	C>T	rs72474224	Nonsyndromic hearing loss and deafness (AR)	1	0/56 (0/62)	0/50 (1/63)
15:28230247	C>T	rs121918166	Tyrosinase-positive oculocutaneous albinism (Unknown)	2	0/56 (0/62)	0/50 (1/63)
16:3293310	A>G	rs28940579	Familial Mediterranean fever| not provided (AR)	2	0/56 (0/62)	1/50 (1/63)
16:8905010	G>A	rs28936415	Carbohydrate-deficient glycoprotein syndrome type I| not provided (AR)	2	0/56 (0/62)	1/50 (1/63)
17:3402294	A>C	rs28940279	Spongy degeneration of central nervous system (AR)	2	0/56 (0/62)	1/50 (1/63)
17:7125591	T>C	rs113994167	Very long chain acyl-CoA dehydrogenase deficiency| not provided (AR)	2	1/50 (1/62)	1/50 (1/63)
18:29178618	G>A	rs76992529	Amyloidogenic transthyretin amyloidosis| not provided (AD)	2	4/50 (4/62)	0/50 (0/63)
18:58039478	G>T	rs13447324	Obesity (AD)	2	0/50 (0/62)	1/50 (1/63)

**Table 3.** Carriers of ClinVar pathogenic variants and *APOL1* renal risk alleles, by return of research results survey responses. Shown are the proportions of pathogenic ClinVar two-star variants and one or two *APOL1* renal risk alleles (RA) by survey response for those participants who were genotyped.

Survey response	% ClinVar pathogenic two-star variant carriers	% *APOL1* RA_1_	% *APOL1* RA_2_
Information about your genes that may influence your doctor’s approach to your care (C only; n=35)	17%	14%	11%
Information about your genes that has uncertain significance and will not change the way that your doctor treats you (D only; n=16)	25%	19%	6%
C and D (n=35)	23%	17%	14%
I do not want to receive any results (E only; n=7)	29%	29%	14%
